# Baseline and usual triglyceride-glucose index and the risk of chronic kidney disease: a prospective cohort study

**DOI:** 10.1007/s11357-023-01044-5

**Published:** 2024-01-05

**Authors:** Setor K. Kunutsor, Samuel Seidu, Sudhir Kurl, Jari A. Laukkanen

**Affiliations:** 1 Real World Evidence Unit, Diabetes Research Centre, University of Leicester, Leicester General Hospital, Gwendolen Road, Leicester, LE5 4WP UK; 2 Department of Medicine, Institute of Clinical Medicine, University of Eastern Finland, Kuopio, Finland; 3 Institute of Public Health and Clinical Nutrition, University of Eastern Finland, Kuopio, Finland; 4 Wellbeing Services County of Central Finland, Department of Medicine, Jyväskylä, Finland

**Keywords:** Triglyceride-glucose index, Chronic kidney disease, Cohort study

## Abstract

Triglyceride-glucose (TyG) index is an emerging marker of adverse cardiometabolic conditions such as cardiovascular disease and type 2 diabetes. The long-term relevance of TyG index to chronic kidney disease (CKD) is uncertain. We aimed to assess the association of TyG index with CKD risk and its utility in risk prediction in a prospective study. The TyG index was calculated using fasting triglycerides and fasting plasma glucose (FPG) levels measured in 2362 men aged 42–61 years with normal kidney function using the formula: Ln (fasting triglycerides [mg/dL] × FPG [mg/dL]/2). Multivariable adjusted hazard ratios (HRs) (95% confidence intervals, CIs) were estimated for CKD. Correction for within-person variability was made using data from repeat measurements of triglycerides and FPG taken 11 years after baseline. Over a median follow-up duration of 17.5 years, 223 CKD cases were recorded. The age-adjusted regression dilution ratio for the TyG index was 0.54 (95% CI, 0.48–0.60). The risk of CKD increased continuously with increasing TyG index across the range 9.3 to 11.6 (*p* value for nonlinearity<.001). In analysis adjusted for established risk factors, a unit higher TyG index was associated with an increased risk of CKD (HR 1.59, 95% CI 1.24–2.05). Comparing extreme tertiles of the TyG index, the corresponding adjusted HR (95% CI) for CKD was 1.61 (1.15–2.27). Addition of the TyG index to a CKD risk prediction model containing established risk factors improved risk discrimination and reclassification (*p* value for difference in −2 log likelihood<.001; NRI=47.66%, *p*=.014; IDI=0.0164, *p*<.001). Higher TyG index is associated with an increased risk of CKD and improves the prediction and classification of CKD beyond established risk factors. Using single baseline estimations of the TyG index to investigate its association with CKD risk could considerably under-estimate the true association.

**Supplementary Information:**

The online version contains supplementary material available at 10.1007/s11357-023-01044-5.

## Introduction

Chronic kidney disease (CKD) is a growing global health concern. Its epidemiology reveals a troubling burden, both in terms of morbidity and mortality, and there are substantial economic costs associated with its diagnosis and management.[1, 2] Multiple modifiable and non-modifiable risk factors including age, gender, hypertension, diabetes, obesity, smoking, and atherosclerotic cardiovascular disease (CVD) are associated with the onset and progression of CKD.[3, 4] Despite extensive efforts to mitigate its spread, CKD prevalence and incidence continue to rise.[2] Hence, it becomes imperative to identify simple, readily accessible risk markers that can help identify individuals at increased risk of developing this debilitating condition.

The triglyceride-glucose index (TyG index) has emerged as a reliable surrogate measure of insulin resistance.[5, 6] It is calculated from fasting triglyceride and glucose levels, offering a convenient and cost-effective means of assessing insulin resistance. In recent times, the TyG index has attracted attention for its association with adverse cardiometabolic conditions, including metabolic syndrome, type 2 diabetes (T2D), coronary artery disease, and cardiovascular disease (CVD).[7–10] Chronic kidney disease shares a close relationship with these adverse cardiometabolic conditions, through shared risk factors and possibly pathophysiological mechanisms. For instance, insulin resistance has been shown to be a critical pathway in the development of these conditions;[11] insulin resistance is also present in the early stages of CKD [12] and may be linked to the progression of CKD. Given the overall evidence, it is reasonable to hypothesize that the TyG index may also be linked to the risk of CKD. A number of studies have reported on the associations between TyG index and the risk of CKD, but the majority of these studies were based on cross-sectional study designs [13, 14] or were conducted in specific populations, such as those with pre-existing disease such as diabetes or hypertension.[13, 15] There is limited evidence on the relationship between TyG index and risk of CKD in approximately general populations. Furthermore, accounting for within-person variability in levels of the TyG index has not been addressed in previous studies. Data on the extent to which an exposure varies within individuals enhances the interpretation of epidemiological studies in an aetiological context. As a result of measurement errors, lifestyle changes, ageing, and development of chronic disease during long-term follow-up, analysis which only employs baseline measurements of the TyG index could underestimate the true strength of any aetiological association between TyG and CKD risk (i.e., “regression dilution bias”[16]). It is possible that previous estimates of the association between the TyG index and CKD risk may have been biased due to inability to correct for regression dilution bias. Therefore, the primary aim of this study was to elucidate the nature, magnitude, and specificity of the association between the TyG index and the risk of CKD using a population-based prospective cohort study based on an approximately general healthy population. Repeat measurements of circulating levels of fasting triglycerides and glucose performed several years apart in a random sample of participants enabled quantification of within-person variability in the TyG index. Finally, we also assessed the extent to which TyG index measurements could improve the prediction of CKD using measures of risk discrimination and reclassification.

## Materials and methods

### Study design and participants

The study was conducted in accordance with STROBE (STrengthening the Reporting of OBservational studies in Epidemiology) guidelines for reporting observational studies in epidemiology (Electronic Supplementary Material 1). The analysis utilized data from the initial Kuopio Ischemic Heart Disease (KIHD) population-based prospective cohort study, which comprised a representative sample of men aged 42–61 years, drawn from eastern Finland and had baseline assessments and physical examinations between March 1984 and December 1989.[17] Details of the study design and recruitment methods have been described previously.[18, 19] Study participants of the first cohort were subsequently re-examined at 4 years, 11 years, and 20 years At the 11-year follow-up examination, women were invited to join the study. This second cohort comprising 1774 men and women, who had baseline examinations carried out between March 1998 and December 2001, had restricted coverage of key covariates and outcomes, making it less suitable for comprehensive analysis compared to the original, larger male cohort. For the first KIHD cohort, of the 3433 representative sample of men invited for screening, 3235 were found to be potentially eligible, and of this number, 2682 provided consent to participate in the study and 553 did not respond to the invitation or declined to participate. For this analysis, we excluded men with (i) existing kidney disease at baseline (*n*=56) and (ii) missing data on the exposure or potential confounders (*n*=264). This left a total of 2362 men with non-missing data on TyG index, covariates, and CKD events (Electronic Supplementary Material 2). The study protocol was approved by the Research Ethics Committee of the University of Kuopio and Kuopio University Hospital, Kuopio, Finland (License number 143/97), and all study procedures were conducted in accordance with the Declaration of Helsinki. Written informed consent was obtained from all participants.

### Assessment of exposure, covariates, and outcome

Physical and blood biomarkers measurements and assessment of lifestyle characteristics and medical history have been previously described.[17, 20] Blood biomarkers, including lipoproteins, were measured in participants who provided blood specimens between 8:00 and 10:00 a.m. after abstaining from alcohol consumption for 3 days, refraining from smoking for 12 h, and fasting overnight. Serum samples were stored frozen at −80 °C before measurements of lipids and biochemical analytes. The cholesterol content of lipoprotein fractions and serum triglycerides were assayed enzymatically using the Boehringer Mannheim method (Mannheim, Germany).[21] Fasting plasma glucose (FPG) was determined using fresh samples, which was measured using the glucose dehydrogenase method (Merck, Darmstadt, Germany) after protein precipitation by trichloroacetic acid. The TyG index was calculated using the formula: Ln (fasting triglycerides [mg/dL] × FPG [mg/dL]/2). In a random subset of participants, triglyceride and FPG levels were re-assessed at 11 years following the baseline measurements, which enabled computation of repeat TyG index. The measurements being limited to a subsample of participants was indeed a constraint dictated by the study design and available resources. Due to practical constraints, it was not feasible to perform these repeat measurements across the entire study population. To manage these limitations, a random subset of participants was selected for these measurements. Body mass index (BMI) was estimated as weight in kilograms divided by the square of height in meters. Lifestyle characteristics, including smoking, alcohol consumption, physical activity, and socioeconomic status (SES), as well as prevalent medical conditions, were assessed using self-administered lifestyle and health questionnaires.[22] Socioeconomic status assessment involved creating a summary index based on indicators such as income, education, occupational prestige, material standard of living, and housing conditions.[23–25] The composite SES index ranged from 0 to 25, with higher values indicating lower SES. A history of coronary heart disease (CHD) was defined as previous myocardial infarction, angina pectoris, the use of nitroglycerin for chest pain ≥ once a week, or chest pain. Energy expenditure from physical activity was assessed using the validated KIHD 12-month leisure-time physical activity questionnaire.[26, 27] Chronic kidney disease (CKD) was defined based on the National Kidney Foundation Kidney Disease Outcomes Quality Initiative (KDOQI) guideline, encompassing kidney damage (e.g., albuminuria) or an estimated glomerular filtration rate (eGFR) lower than 60 mL/min per 1.73 m2 (or both) for a duration of 3 months or longer.[28] Incident CKD cases that occurred from the study’s commencement until 2014 were included in the analysis.[17, 29, 30] All CKD events were identified by computer linkage to the National Hospital Discharge Registry data. Each event was validated by two physicians who were blinded to the exposures following detailed cross-checking of medical documents.

### Statistical analysis

Skewed variables (alcohol consumption and physical activity) were log transformed to achieve approximately normal distributions. Baseline characteristics were reported as means (standard deviation, SD) or median (interquartile range, IQR) for continuous variables and numbers (percentages) for categorical variables. Time-to-event analyses were performed using Cox proportional hazard regression models after confirmation of no major departure from the proportionality of hazards assumptions using scaled Schoenfeld residuals.[31]. We quantified and corrected for within-person variability in the TyG index, which is the extent to which an individual’s TyG index measurements vary around the long-term average exposure levels (“usual levels”). [32] This involved estimating an age-adjusted regression dilution ratio (RDR) by regressing available repeat measurements of the TyG index on baseline values.[33] The RDR assumes that the “usual levels” of the TyG index represent the true long-term exposure of the TyG index on CKD risk. To correct for regression dilution bias, the estimated disease association (log hazard ratio and its 95% confidence intervals) was divided by the RDR.

To explore a potential nonlinear dose-response relationship between the TyG index and CKD risk, we constructed a multivariable restricted cubic spline (RCS) with knots at the 5th, 35th, 65th, and 95th percentiles of the distribution of the TyG index as recommended by Harrell [34]. The TyG index was modelled continuously (per unit increase) and as categories (tertiles) defined according to the baseline distribution of its levels. The adjustment for confounders were based on three models: (Model 1) age; (Model 2) Model 1 plus systolic blood pressure (SBP), total cholesterol, smoking, prevalent T2D, hypertension and CHD, alcohol consumption, SES, eGFR, and physical activity; and (Model 3) Model 2 plus a potential mediator BMI. These covariates were selected based on the following: (i) their established roles as risk factors for CKD [3, 4], (ii) published associations with CKD in the KIHD study,[17, 29, 30, 35] and (iii) their potential as confounders based on known associations with CKD outcomes and observed associations with the exposure using the available data.[36] Formal tests of interaction were used to assess statistical evidence of effect modification by categories of pre-specified clinically relevant individual level characteristics. To investigate whether adding information on the TyG index to traditional risk factors for CKD is associated with improvement in the prediction of CKD risk, we calculated measures of discrimination and reclassification. For discrimination, we tested for differences in the −2 log likelihood of prediction models with and without inclusion of the TyG index. The −2 log likelihood test has been recommended as a more sensitive risk discrimination method.[37, 38] Reclassification was assessed using the net-reclassification-improvement (NRI)[39, 40] and integrated-discrimination-improvement (IDI)[39] by comparing the model containing traditional risk factors to the predicted risk from the model containing traditional risk factors plus the TyG index. All statistical analyses were performed using Stata version MP 18 (Stata Corp).

## Results

### Baseline characteristics and within-person variability in the TyG index

Table 1 summarizes the baseline characteristics of the 2362 participants overall and by CKD development at end of follow-up. At study entry, their mean ±SD age was 53 ±5 years, while the mean ±SD TyG index was 8.5 ±0.6. Participants in the top tertile of TyG index had higher levels of SBP, BMI, total cholesterol, and physical activity, smoked less, and more likely to have comorbidities such as T2D, hypertension, and CHD (Table 2). In a random subset of 726 participants who had triglyceride and FPG levels re-assessed at 11 years following the baseline measurements, the mean ±SD of repeat TyG index was 8.5 ±0.5. Overall, the age-adjusted RDR of the TyG index was 0.54 (95% CI: 0.48 to 0.60), which suggests that the association of the TyG index with CKD risk using baseline measurements of this index could under-estimate the true association by [(1/0.54)−1]*100 = 85.2%.

**Table 1:** Baseline characteristics of study participants overall and by chronic kidney disease

Characteristics	Overall (*n*=2362) Mean ±SD or median (IQR)	With CKD (*n*=223) Mean ±SD or median (IQR)	Without CKD (*n*=2139) Mean ±SD or median (IQR)
TyG index	8.5 ±0.6	8.6 ±0.7	8.5 ±0.6
Age, yr	53 ±5	54 ±4	53 ±5
Socioeconomic status	8.6 ±4.2	9.5 ±4.2	8.5 ±4.2
Systolic blood pressure, mmHg	134 ±17	138 ±18	134 ±17
Body mass index, kg/m^2^	26.9 ±3.6	27.9 ±4.0	26.8 ±3.5
Alcohol consumption, g/week	31.8 (6.3, 94.0)	30.5 (5.4, 97.4)	32.0 (6.4, 93.8)
Physical activity, KJ/day	1181 (621, 1964)	1236 (593, 1900)	1177 (623, 1977)
Current smoking	755 (32.0%)	63 (28.3)	692 (32.4%)
History of type 2 diabetes	95 (4.0%)	12 (5.4%)	83 (3.9%)
History of hypertension	716 (30.3%)	80 (35.9%)	636 (29.7%)
History of coronary heart disease	598 (25.3%)	76 (34.1%)	522 (24.4%)
Total cholesterol, mmol/l	5.92 ±1.10	5.97 ±1.13	5.91 ±1.09
Estimated GFR, ml/min/1.73 m^2^	87.9 ±16.5	87.3 ±15.6	88.0 ±16.5

Socioeconomic status was generated as a summary index that combined factors such as income, education, occupational prestige, material standard of living, and housing conditions. The composite index ranged from 0 to 25, with higher values indicating lower socioeconomic status

*CKD* chronic kidney disease, *GFR* glomerular filtration rate, *IQR* interquartile range, *SD* standard deviation, *TyG* triglyceride-glucose

**Table 2:** Baseline characteristics of study participants by tertiles of TyG index

Characteristics	TyG index		
	Tertile 1 (*N*=885) Mean ±SD or median (IQR)	Tertile 2 (*N*=820) Mean ±SD or median (IQR)	Tertile 3 (*N*=657) Mean ±SD or median (IQR)
Age, yr	53 ±5	53 ±5	53 ±5
Socioeconomic status	8.6 ±4.3)	8.8 ±4.3	8.4 ±4.1
Systolic blood pressure, mmHg	131 ±16)	134 ±17	138 ±17
Body mass index, kg/m^2^	25.5 ±3.0	27.1 ±3.4	28.5 ±3.8
Alcohol consumption, g/week	32.4 (6.4, 92.1)	26.7 (5.0, 81.8)	37.1 (7.4, 112.1)
Physical activity, KJ/day	1233 (680, 1985)	1087 (573, 1865)	1257 (621, 2118)
Current smoking	280 (31.6%)	275 (33.5%)	200 (30.4%)
History of type 2 diabetes	9 (1.0%)	19 (2.3%)	67 (10.2%)
History of hypertension	181 (20.5%)	255 (31.1%)	280 (42.6%)
History of coronary heart disease	176 (19.9%)	203 (24.8%)	219 (33.3%)
Total cholesterol, mmol/l	5.57 ±0.99	6.02 ±1.09	6.27 ±1.11
Estimated GFR, ml/min/1.73 m^2^	88.9 ±15.0	88.0 ±15.6	86.4 ±19.0

Socioeconomic status was generated as a summary index that combined factors such as income, education, occupational prestige, material standard of living, and housing conditions. The composite index ranged from 0 to 25, with higher values indicating lower socioeconomic status

*CKD* chronic kidney disease, *GFR* glomerular filtration rate, *IQR* interquartile range, *SD* standard deviation, *TyG* triglyceride-glucose

### TyG index and risk of CKD

**Fig. 1:**
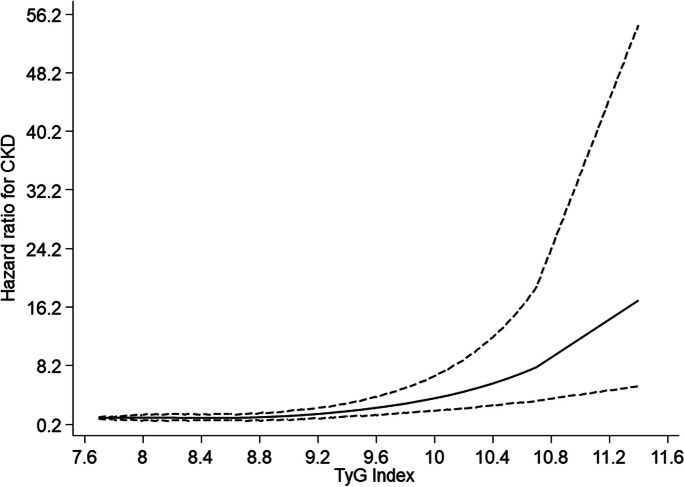
Restricted cubic splines of the hazard ratios of chronic kidney disease with baseline TyG index. CKD chronic kidney disease, TyG triglyceride-glucose. Dashed lines represent the 95% confidence intervals for the spline model (solid line). Models were adjusted for age, systolic blood pressure, total cholesterol, smoking, prevalent type 2 diabetes, hypertension and coronary heart disease, alcohol consumption, socioeconomic status, estimated glomerular filtration rate, and physical activity

**Fig. 2:**
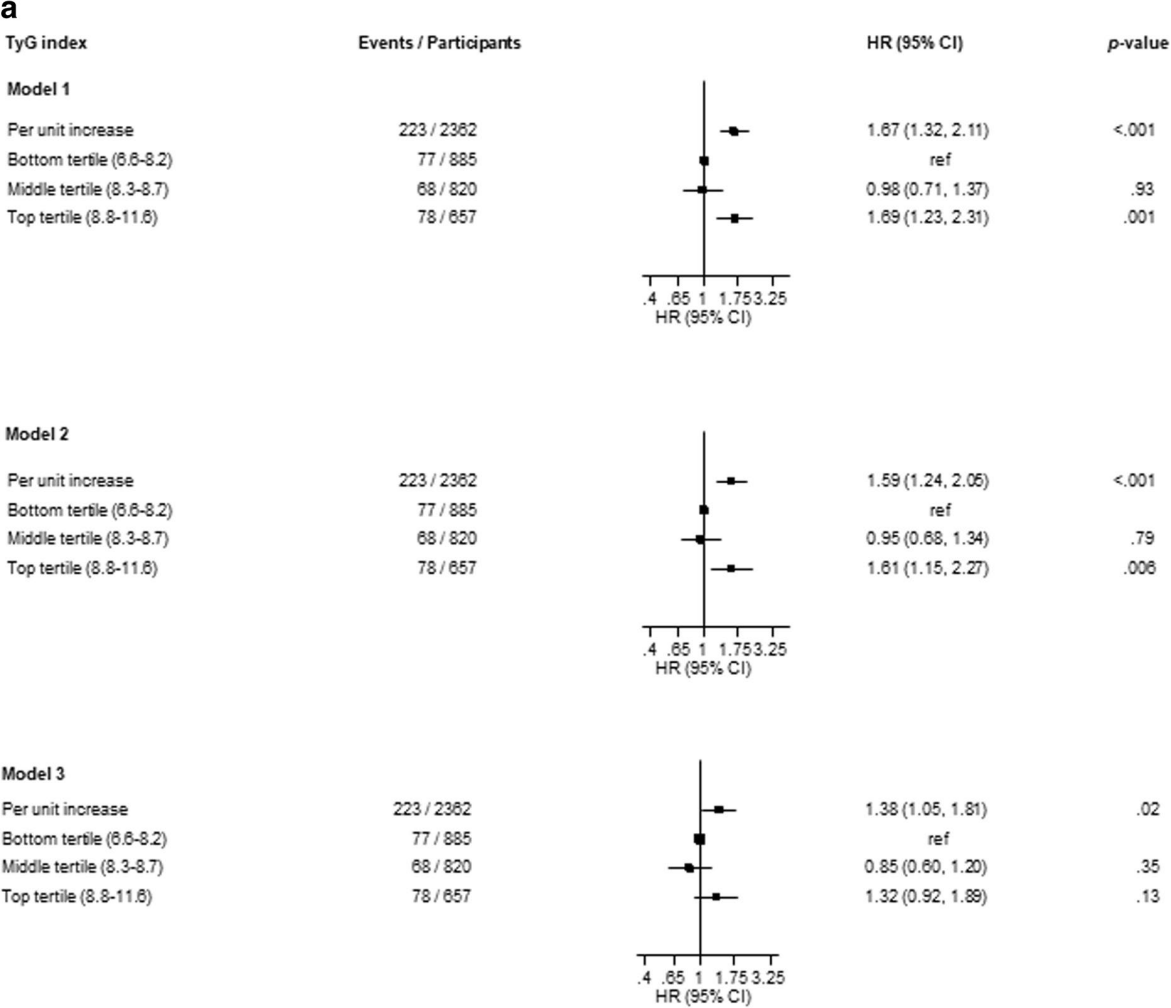

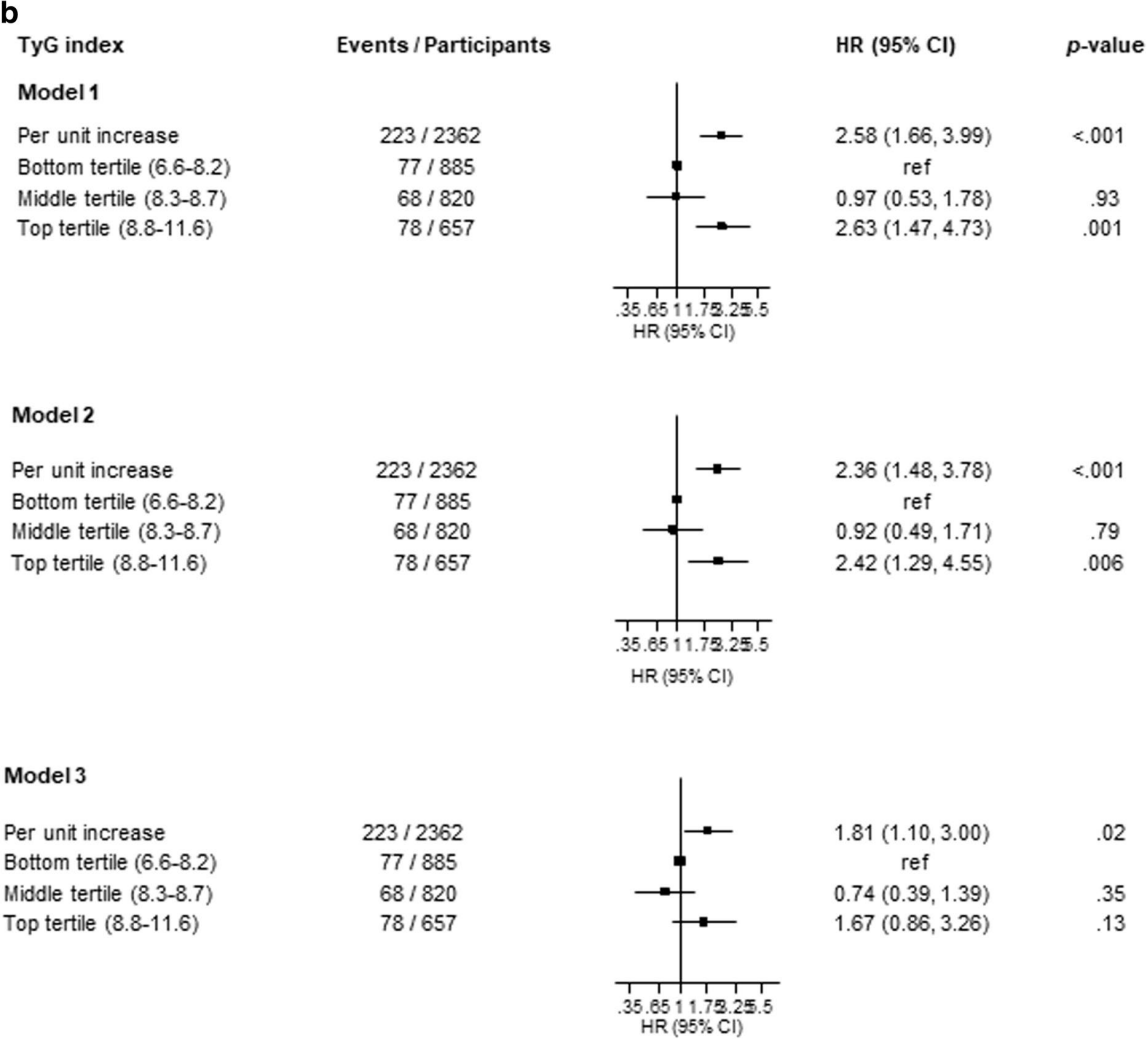
Association of TyG index with chronic kidney disease. (**A**) Using baseline levels of TyG index (**B**). Using usual levels of TyG index (corrected for regression dilution bias). ref reference, TyG triglyceride-glucose. Model 1: Adjusted for age. Model 2: Model 1 plus systolic blood pressure, total cholesterol, smoking, prevalent type 2 diabetes, hypertension and coronary heart disease, alcohol consumption, socioeconomic status, estimated glomerular filtration rate, and physical activity. Model 3: Model 2 plus body mass index

**Fig. 3:**
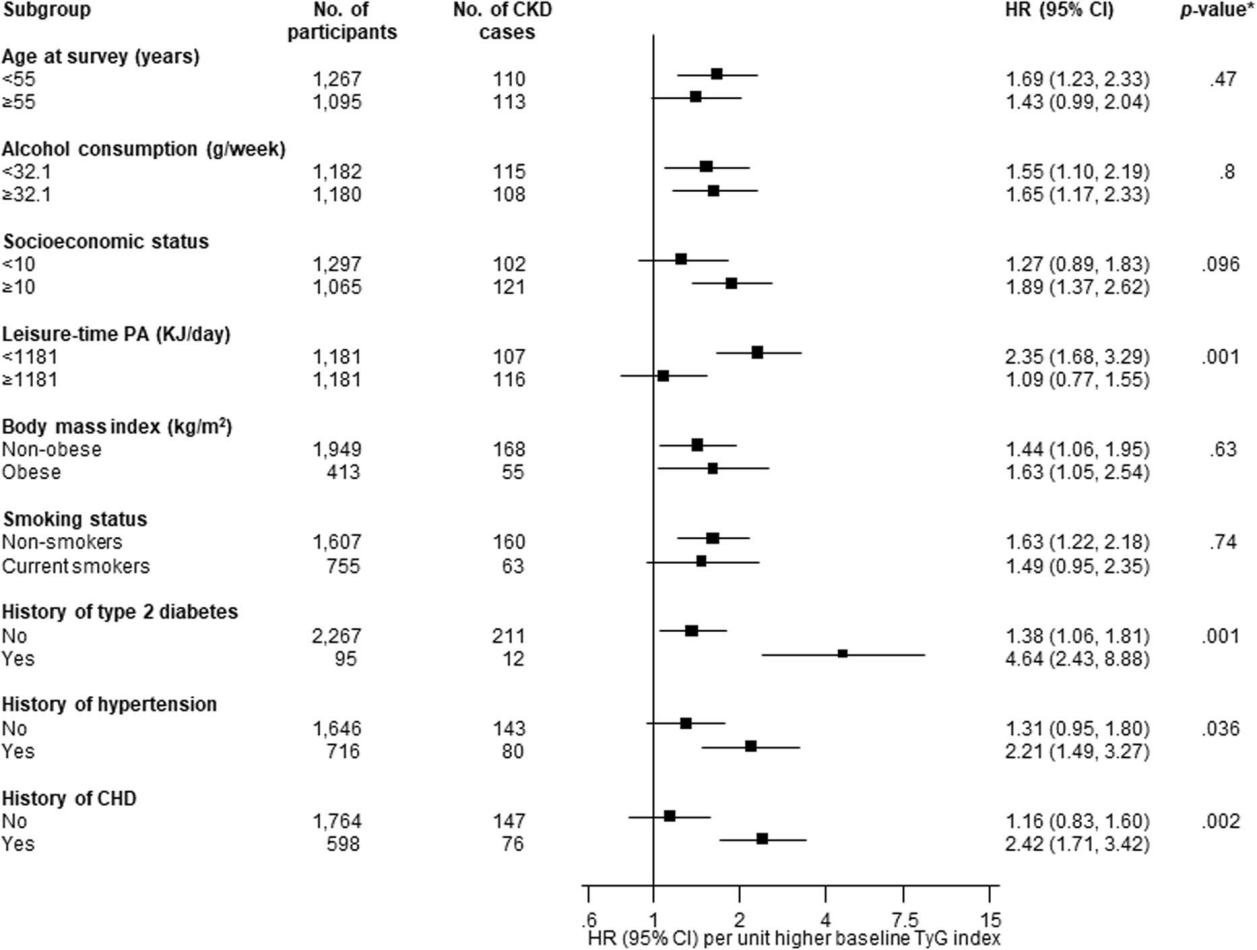
Hazard ratios for baseline values of TyG index and chronic kidney disease risk by several participant level characteristics. Hazard ratios are adjusted for age, systolic blood pressure, total cholesterol, smoking, prevalent type 2 diabetes, hypertension and coronary heart disease, alcohol consumption, socioeconomic status, estimated glomerular filtration rate, and physical activity; CHD coronary heart disease, CI confidence interval, CKD chronic kidney disease, HR hazard ratio, PA physical activity, SBP systolic blood pressure, TyG triglyceride-glucose. *, *p* value for interaction; cutoffs used for age, alcohol consumption, socioeconomic status, and physical activity are median values

After a median (IQR) follow-up duration of 17.5 (25.5, 27.8) years, a total of 223 CKD cases were recorded. A multivariable RCS curve showed that CKD risk increased continuously with increasing TyG index across the range 9.3 to 11.6 (*p* value for nonlinearity<.001) (Fig. 1). In age-adjusted analysis, each 1 unit increase in TyG index was associated with a significantly increased risk of CKD (HR 1.67, 95% CI 1.32–2.11) (Fig. 2A-Model 1), which was minimally attenuated to (HR 1.59, 95% CI 1.24–2.05) on further adjustment for SBP, total cholesterol, smoking, prevalent T2D, hypertension and CHD, alcohol consumption, SES, eGFR, and physical activity (Fig. 2A-Model 2). The association was further attenuated but remained significant on accounting for BMI (HR 1.38, 95% CI 1.05–1.81) (Fig. 2A-Model 3). Alternatively, comparing individuals in the top versus bottom tertiles of the Tyg index, the corresponding adjusted HRs (95% CIs) for CKD were 1.69 (1.23–2.31), 1.61 (1.15–2.27) and 1.32 (0.92–1.89), respectively (Fig. 2A). On correction for regression dilution bias, the HRs were stronger (Fig. 2B). In interaction analysis, there was evidence of effect modification by physical activity level and prevalent T2D, hypertension and CHD (*p value* for interaction <.05 for each; Fig. 3). The –2 log likelihood was significantly improved on addition of the TyG index to the risk model containing established risk factors for CKD (*p* for comparison<.001). The continuous NRI and IDI were 47.66% (95% CI: 9.69 to 85.63; *p*=.014) and 0.0164 (0.0082 to 0.0247; *p*<.001), respectively.

## Discussion

In this population-based cohort of middle-aged and older Caucasian men, higher TyG index was associated with an increased risk of CKD. A dose-response curve showed a potential nonlinear relationship between the TyG index and CKD risk; however, the risk of CKD increased gradually and continuously with increasing TyG index across the range 9.3 to 11.6. When TyG index was modeled as a continuous variable, the association was independent of several potential confounders as well as the potential mediator BMI. However, when TyG index was modeled as a categorical variable, the association was partly dependent on BMI, which could be attributed to the loss of statistical power because of the categorization. Furthermore, if BMI is truly a mediator, then adjusting for it constitutes an overadjustment. Subgroup analyses showed that the association between higher TyG index and increased CKD risk was more pronounced in those with lower physical activity levels compared to higher physical activity levels and those with a history of T2D, hypertension, or CHD compared to those without a history of any of these comorbidities. On accounting for regression dilution bias, our results showed that using single baseline measurements of the TyG index to investigate its association with CKD risk could under-estimate the true association by as high as 85%. With regard to the potential utility of TyG index measurements for CKD risk assessment, the addition of information on the TyG index to a risk model containing traditional risk factors for CKD was associated with significant improvements in the discrimination and reclassification of long-term CKD risk.

Though a number of prospective cohort studies have reported on the associations between the TyG index and CKD risk,[41] certain relevant aspects of the association were not addressed by these studies. These included (i) the detailed dose-response nature of the relationship; (ii) accounting for within-person variability in values of the TyG index; (iii) investigating if clinically relevant risk markers could modify the association; and (iv) assessing if information on the TyG index could improve CKD risk prediction using formal risk prediction analyses. Ren and colleagues [41] recently reported on a comprehensive investigation on the relationship between the TyG index and CKD risk using a cohort analysis and meta-analysis of previous studies. Their primary cohort analysis showed that higher TyG index was associated with an increased risk of CKD, independently of established risk factors. The relationship was consistent with a linear dose-response relationship; CKD risk increased continuously with increasing TyG index across the range 8.0 to 12.0, findings which were qualitatively similar to ours. Their meta-analysis included a mix of cross-sectional and cohort studies as well as studies based on participants with specific diseases.[41] The novelty of our study was the ability to account for long-term changes in TyG index values and assess the value of the TyG index in long-term CKD risk prediction.

Several factors may contribute to the link between the TyG index and CKD risk, shedding light on the potential mechanisms at play. The TyG index, derived from fasting triglyceride and glucose levels, has consistently demonstrated its reliability as a marker of insulin resistance.[5, 6] Persistent insulin resistance is recognized as a significant contributor to the development of various chronic diseases, including T2D, CVDs, [7–10] and now, as our study suggests, CKD. This underscores the importance of insulin resistance as a central mechanistic pathway bridging the TyG index and CKD risk. Insulin resistance disrupts normal glucose metabolism, resulting in chronic hyperglycemia. This, in turn, contributes to abnormal lipid metabolism, culminating in chronic inflammation, oxidative stress, and endothelial dysfunction.[42] These adverse metabolic consequences can set the stage for kidney damage. The chronic inflammation and oxidative stress are known contributors to CKD progression,[43] further emphasizing their potential role in the association between the TyG index and CKD risk. Hyperinsulinemia, a hallmark of insulin resistance, can induce renal vasodilation,[44] leading to impairment of glomerular hyperfiltration and subsequently the development and progression of CKD. While insulin resistance emerges as a central player in this association, it is important to recognize the multifaceted nature of CKD development. The findings of more pronounced associations between a higher TyG index and increased CKD risk in individuals with lower physical activity levels (sedentary behaviour) or a history of T2D, hypertension, or CHD is expected and likely reflects the fact that these risk factors amplify kidney damage when combined with renal stressors linked to a higher TyG index. Further research is warranted to elucidate the precise mechanisms through which the TyG index contributes to CKD, potentially opening avenues for targeted interventions to mitigate this risk.

The findings have implications for CKD prevention and management. The TyG index, calculated from commonly available laboratory measurements, emerges as a practical and accessible tool for routine clinical practice. Its simplicity and ease of measurement make it an attractive candidate for inclusion in regular health assessments. Early detection of CKD risk is paramount in preventing disease progression and implementing timely interventions. The TyG index’s utility in stratifying individuals at higher risk of CKD provides healthcare providers with a potential additional valuable tool to identify susceptible individuals at an earlier stage. This proactive approach can facilitate personalized patient care, including lifestyle modifications and intensified monitoring, which may help mitigate CKD risk and its associated complications and overall burden. Further research and validation studies are warranted to replicate these findings in other populations.

Other strengths in addition to those listed previously include the population-based prospective cohort design, the long-term follow-up which was adequate for the ascertainment of outcomes, and the comprehensive analysis, which provides more detailed insights on the relationship between the TyG index and CKD. However, the limitations deserve consideration. First, the study population consisted of middle-aged and older Finnish men, which limits the generalizability of the findings to women and other populations. Indeed, future research could aim to replicate and extend our findings in a more diverse population to validate and broaden the applicability of our conclusions. Second, there is a potential for biases such as residual confounding and reverse causation given the observational cohort design. Third, we had no data on the precise cause of CKD and its classification. Finally, detailed evaluation of within-person variability in levels of TyG index could not be done given that repeat measurements were only available in a subset of participants; this also prevented the conduct of a time-varying analysis. Hence, the results may not fully encapsulate the variability present in the entire cohort.

## Conclusion

Findings from a new prospective cohort study show that higher TyG index is associated with an increased risk of CKD and improves the prediction and classification of CKD beyond common established risk factors.

Using single baseline estimations of the TyG index to investigate its association with CKD risk could considerably under-estimate the true association.

### Supplementary information

ESM 1 (DOCX 66 kb)
